# Deaths

**Published:** 1915-02

**Authors:** 


### Deaths.

Dr. J. J. Atkins died at his home in Refugio, Tuesday, December 12.

Dr. T. L. Keys of Rockwall died January 11.

Dr. Bascomb Kavanaugh of San Augustine died January 15.

Dr. J. E. Cooper of Golden died January 19.

Dr. C. F. Yeager of Mineral Wells died January 19.

Dr. L. G. Bombarger of Fort Worth died January 15.

Dr. H. G. Thomas of Handley, Texas, died January 1.

Dr. P. C. Woodruff of Roxton died January 7.

Dr. C. B. Merchant of Quinlan, Texas, died December 29.

